# In vitro wound healing potential of cyclohexane extract of *Onosma dichroantha* Boiss. based on bioassay-guided fractionation

**DOI:** 10.1038/s41598-023-31855-7

**Published:** 2023-03-28

**Authors:** Fereshteh Safavi, Mahdi Moridi Farimani, Masoud Golalipour, Houman Bayat

**Affiliations:** 1 Department of Chemistry, Faculty of Science, Golestan University, Gorgan, Iran; 2 Department of Phytochemistry, Medicinal Plants and Drug Research Institute, Shahid Beheshti University, Evin, 1983969411 Tehran Iran; 3 Medical Cellular and Molecular Research Center, Golestan University of Medical Sciences, Gorgan, Iran; 4 Niak Pharmaceutical Company, Golestan, Gorgan, Iran

**Keywords:** Secondary metabolism, Natural products

## Abstract

*Onosma dichroantha* Boiss. is a biennial herb used in traditional medicine in Iran for healing wounds and burns. Our previous study demonstrated that cyclohexane extract of *O. dichroantha* Boiss. enhanced wound healing in vitro. The aim of the present study was to identify the active fractions and compounds responsible for this effect through bio-guided fractionation followed by three in vitro tests for anti-inflammation, proliferation, and migration (scratch test). Fractionation of the CE extract yielded six fractions (Fr. A to Fr. F). Fr. F showed the most remarkable wound healing activity in three assays. Fr. F was further fractionated into five subfractions (FF-SUB1 to FF-SUB5). FF-SUB1 and FF-SUB2 were selected for further purification based on their wound healing activity. The major components, F. F1 to F. F5, were isolated from these two subfractions and identified as acetylshikonin, deoxyshikonin, β, β-dimethylacrylshikonin, β-hydroxyisovalerylshikonin, and *trans*-anethole of the active subfractions. Bioassay-guided fractionation revealed that naphthoquinone derivatives, as an active component, are responsible for the wound healing properties of the fractions and subfractions of cyclohexane extract of *O. dichroantha* roots. The findings indicate that these fractions and subsections, as well as purified compounds, have a high potential for further investigation as an effective therapeutic agent in wound healing using in vivo models.

## Introduction

To date, about 100 genera and more than 2000 species have been identified in the Boraginaceae family, most of which are distributed in tropical and temperate regions^1^. The genus Onosma is one of the most significant in this family and includes about 230 species^2^. This genus has 47 perennial herbaceous species in Iran. Other species grow in Iraq, Syria, Palestine, Anatolia, the Caucasus, Pakistan and Armenia^3^. Previous studies have shown that the genus Onosma contains phenolic compounds, naphthoquinones, and alkaloids^4^. Alkanine and shikonine are natural compounds with the structure of isohexnylnaphthazene. This chiral pair is found in many genera of the Boraginaceae family, Echium, Alkanna, Onosma, Lithospermum, and Arnebia^5^.

*Onosma dichroantha* Boiss. is known as "Hava Choobeh" in Iran. People traditionally used the extract of this plant to heal wounds and burns. The plant is a biennial herb with a height of 20–50 cm, covered with long, wavy and whitish fibers. The geographical distribution of this species in Iran is in the northern, western and southwestern regions, including Mazandaran, Isfahan, Lorestan, Ilam, Fars and Kerman provinces. They usually grow on sandy cliffs in dry and sunny habitats^6^.

Skin is the first line of protection against infections. That's why it's so important to heal wounds on the skin^7^. Healing a skin wound is a complex and dynamic process with three overlapping phases: inflammation, proliferation, and maturation, which is commonly referred to as remodeling^8^. This well-organized process serves to clear pathogens and cellular debris through the influx of cytokines and growth factors at the wound site immediately after homeostasis. Then, the proliferation of granulation tissue occurs through angiogenesis and immigration of keratinocytes and fibroblasts to the wound site. The maturation phase involves the restoration of the skin barrier and the repairing of the granulation tissue within the scar, along with vessel regression^9^. This newly formed tissue matures over time with greater tensile strength^10^. Although collagen production gradually decreases after three weeks, cross-linking and reorganization of collagen occurs in the last phase of healing for months after the jury is out^11^. There are several factors that affect one or more phases of the wound healing process, and the steps of each phase must be performed accurately and regularly. Interruptions or prolongations of the process can lead to the development of chronic wounds or delayed wound healing. Most chronic ulcers are associated with ischemia, diabetes, and hypertension^12^. Although the healing process in wounds begins and progresses naturally, the outcome is not always favorable^13^. Nowadays, to improve the quality and speed of the healing process, synthetic drugs such as nitrofurazone, gentamicin, mupirocin, and others are used, but these drugs have side effects. Therefore, in recent years, natural medicines have become increasingly used^14^. There are many medicinal plants with a long history of medicinal effects on various diseases. However, there is no scientific information or data for the use of these herbs to prove their effectiveness, or knowledge about the possible active ingredients or mechanism of action. The only basis for their use is tradition^15^.

Cell culture is one of the most widely used techniques in cellular and molecular biology. This is because it allows researchers to study the biology, biochemistry, physiology, and metabolism of both healthy and diseased cells. Primary cells, transformed cells, and self-renewing cells are the three types of cells cultured in the lab, and they are used for various in vitro tests. Cell culture techniques are critical in the development of biotechnology products because these tests are often the quickest to set up and have a wide range of in vitro tests like proliferation, migration, viability, inflammation, and apoptosis^16^.

Both the antibacterial and antioxidant activities of some extracts from the roots of *O. dichroantha* have been investigated using three different methods. Based on the results, all extracts showed antimicrobial properties, especially the acetone extract for Gram-positive bacteria, as well as optimal antioxidant properties by the DPPH method^17^. There are also some reports of hepatitis inhibitory effects on PLC/PRF/5 cells, anti-neuropathic and antidiabetic effects of the *O. dichroantha* in mice^18,19^. Although it has long been used in traditional therapies, it has conducted few phytochemical and biological studies *O. dichroantha*.

In our previous study, we investigated the wound healing effects of three extracts of *O. dichroantha* root, namely cyclohexane, ethyl acetate and methanol. The results showed that cyclohexane extract was the most effective in three in vitro mechanisms of wound healing. Moreover, cyclohexane extract was able to induce angiogenesis in zebrafish^20^.

Based on previous research, we evaluated the wound healing activity of cyclohexane extract of *O. dichroantha* Boiss. using bioassay-guided fractionation procedures in order to identify the active fractions and compounds responsible for this effect. For this purpose, we performed bio-guided fractionation followed by in vitro tests for anti-inflammation, proliferation, and migration (scratch test).

## Results

Based on a previous study^20^, the extract of CE was selected for further fractionation to obtain six fractions. Then, two steps of the experiment were carried out, each of which included a fractionation process and subsequent biological tests. Anti-inflammatory, fibroblast proliferation and migration assay in step I (for six fractions) and anti-inflammatory and fibroblast proliferation assay in step II (for five subfractions) were used for evaluation of wound healing effect as in vitro models. Finally, purification and elucidation of the chemical structure of the main compounds from the effective subfractions were carried out.

### Step 1: Fractionation of the CE extract and bioassay of the obtained fractions

The CE extract was fractionated using the chromatographic method, resulting in six fractions that were designated as Fr. A to Fr. F.

#### In vitro anti-inflammatory study

**Figure 1:**
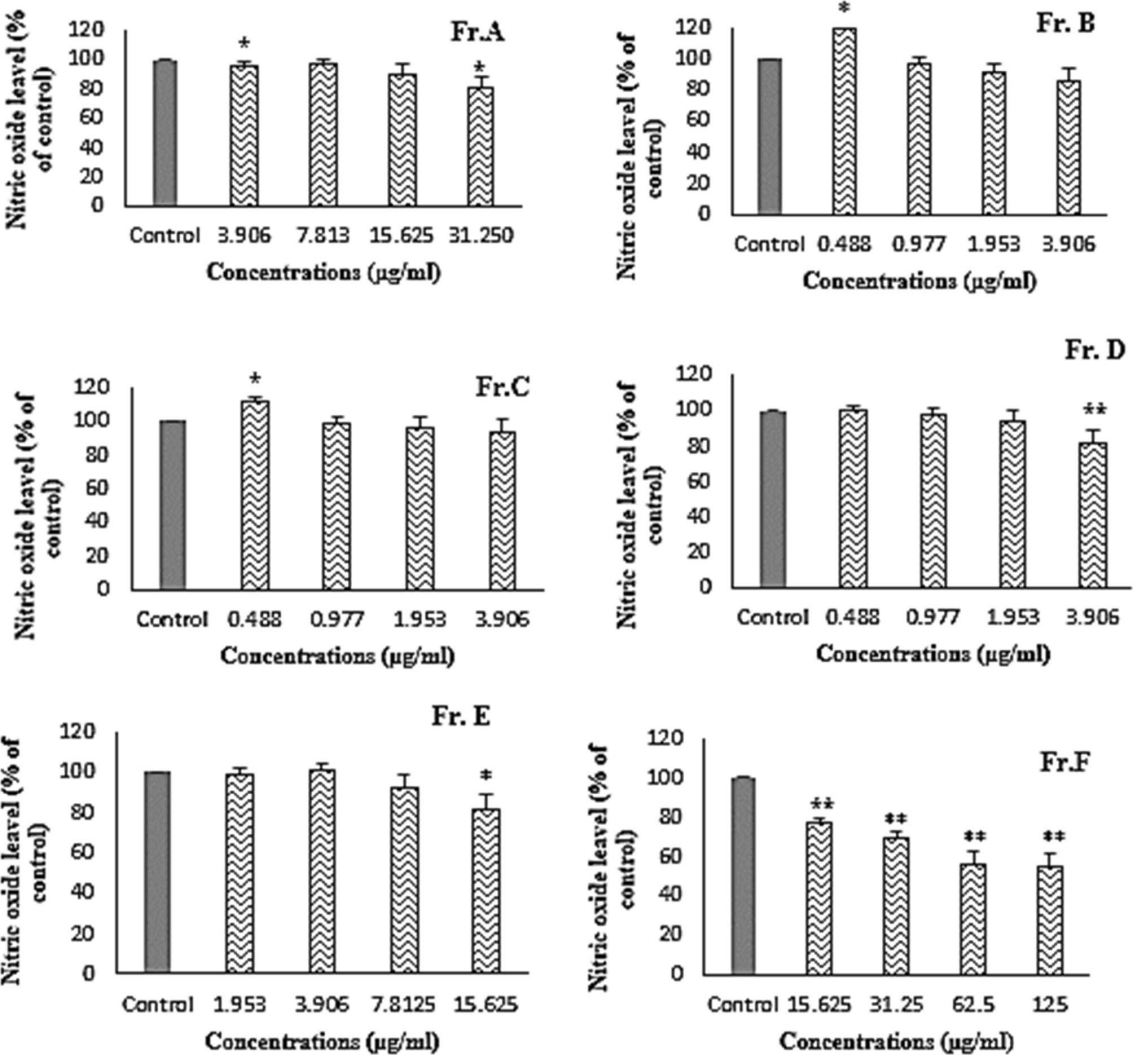
Effect of Fr. A to Fr. F obtained from the cyclohexane extract on nitric oxide production by RAW 264.7 cells after treatment with LPS. All bars and values represent the mean ± SD of three replicate experiments. *Significant difference (P < 0.05) and **P < 0.01 when compared to control group. Non-treated cells are considered as control.

Treatment of cells with Fr. B, Fr. C, and Fr. D for 24 h at the indicated concentrations had cytotoxic effects on RAW 264.7 cells, so the highest concentration used for these three fractions was 3.906 µg/mL. Fr. C was ineffective. Fr. A, Fr. D, and Fr. E suppressed 19.7 ± 5.3%, 18.8 ± 0.8%, and 18.2 ± 6.1% of the NO production, respectively, whereas Fr. F, the most effective fraction at 62.5 µg/mL decreased the production of NO in cells by 43.7 ± 2.1%. When the concentration was increased to 125 g/mL, similar efficacy was observed (with approximately 45.8 4.3% inhibition with P < 0.01 when compared to the control group) (Fig. 1).

#### Fibroblast proliferation assay

**Figure 2:**
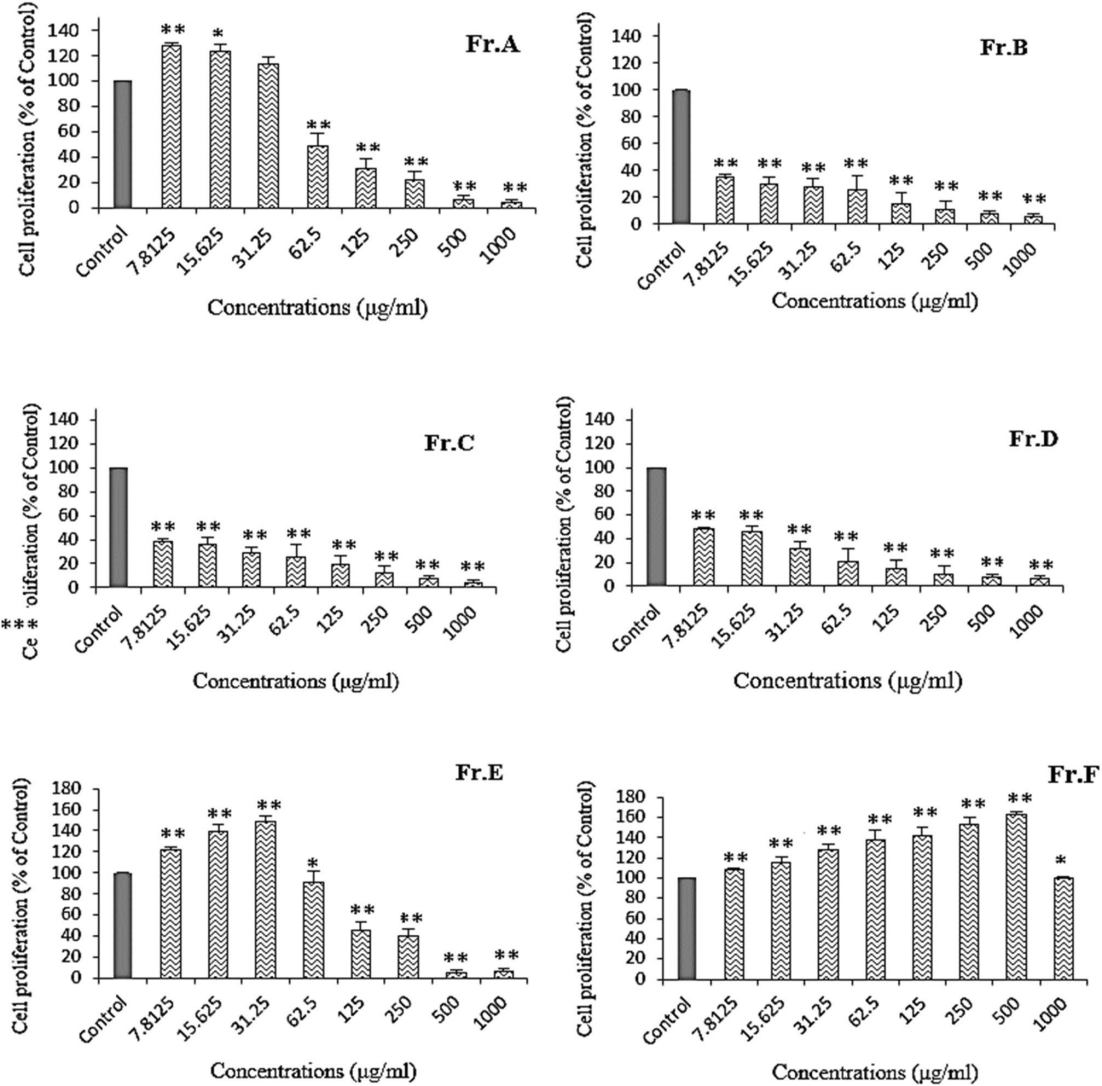
Effect of Fr. A to Fr. F obtained from the cyclohexane extract on the proliferation rate of Hs.27 fibroblast cells. All bars and values represent the mean ± SD of the three replicate experiments. *Significant difference (P < 0.05) and **P < 0.01 when compared to the control group.

Of the six fractions tested, Fr. F and Fr. E were the most potent in stimulating fibroblast proliferation (Fig. 2). Their effective concentrations ranged from 7.81 to 500 μg/mL for Fr. F and from 7.81 to 31.25 μg/mL of Fr. E. Fr. F at 500 μg/mL was able to significantly increase the proliferation of Hs.27 by approximately 62%.

Although Fr. A at concentrations of 7.81–15.61 μg/mL also increased the cell proliferation rate of HS.27 fibroblasts, this increase was less than that of Fr. E and Fr. F (From our results on Fr. A and Fr. E, a decrease in viability of cells was detected at the higher concentrations). The other concentrations and fractions did not show any stimulatory effects at the tested concentrations.

#### Cell migration assay

**Figure 3:**
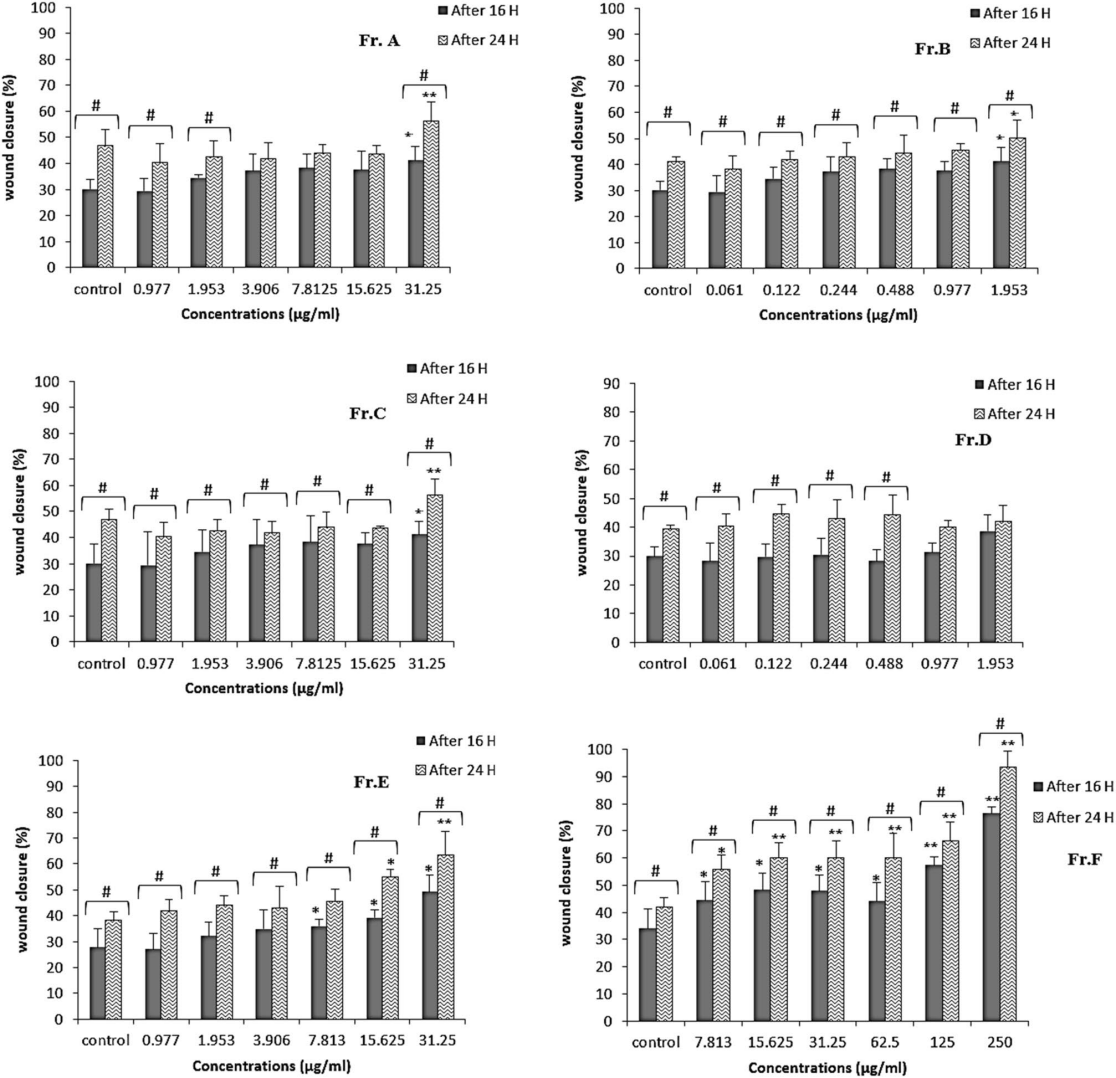
Effect of Fr. A to Fr. F obtained from the cyclohexane extract on the migration rate of scratched HMEC-1 cells. All bars and values represent the mean ± SD of at least three replicate experiments. *P < 0.05 and ** P < 0.01 were considered significant compared to the control group. ^#^P < 0.05 were considered significant to compare the effect of similar concentrations on wound closure at 16 and 24 h by Student's *t*-test.

**Figure 4:**
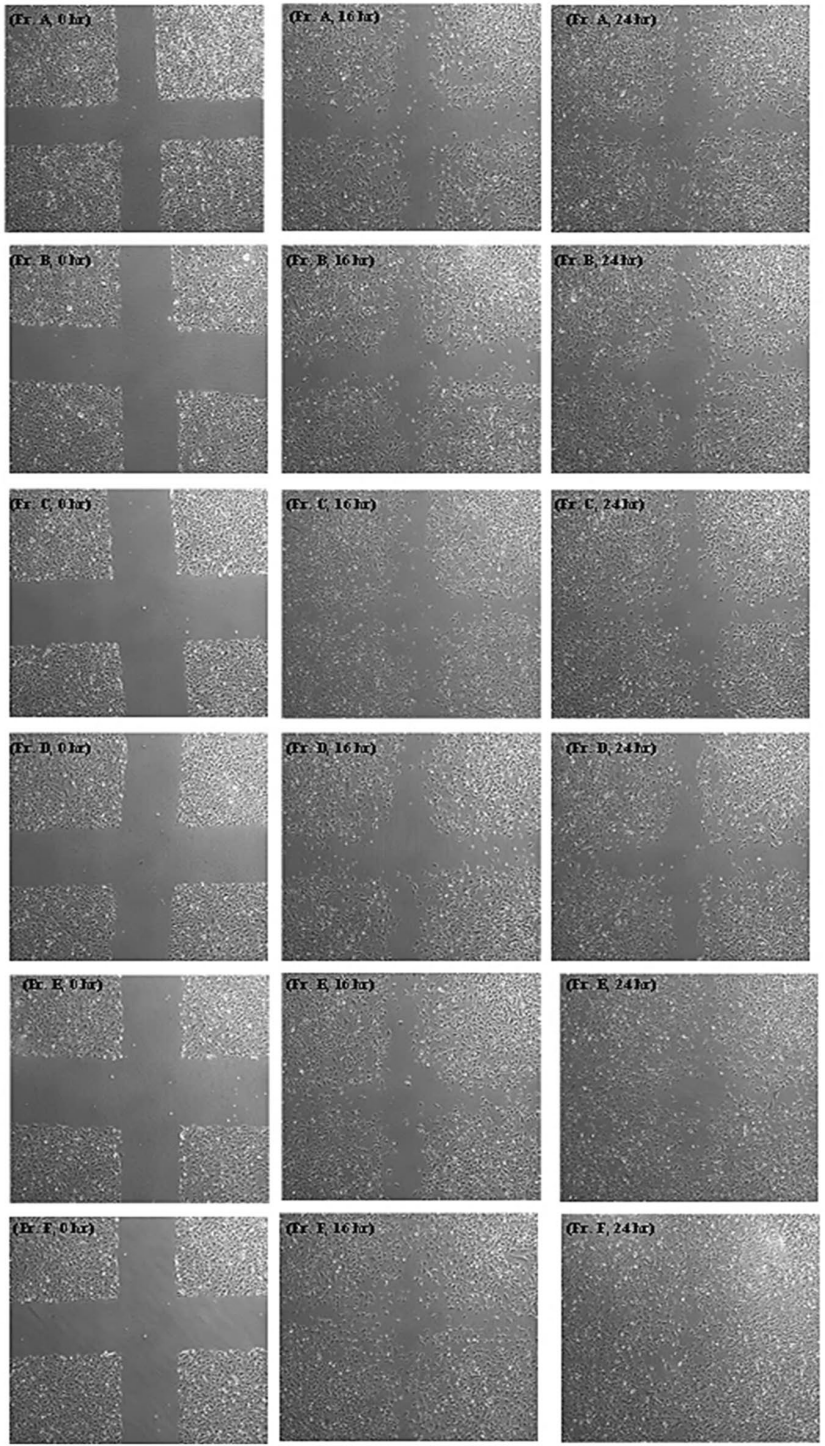
Effect of different fractions obtained from the cyclohexane extract at their maximum non-toxic concentrations on HMEC-1 migration (scratch test). Images from the top to the bottom of the panel: Fr. A, Fr. B, Fr. C, Fr. D, Fr. E and Fr. F respectively. Images were acquired 0, 12, and 24 h after scratching and treatment with the fractions using a fluorescence microscope at 4× magnification. T scratch software was used to analyze the images.

Endothelial cell migration rates were measured in fractions 16 and 48 h after in vitro wound induction. The results of the scratch test showed that fractions Fr. A, Fr. B, Fr. C and Fr. E only in some concentrations had a significant difference from the control group and the rate of wound closure was not significant in these fractions. For Fr. D, P-value showed there was no statistically significant difference with the control group at the tested concentrations. Among the fractions, the highest rate of cell migration was observed in the HMEC-1 cells treated with fraction Fr. F. Compared with the control group, which caused 34 and 42% wound closure at 16 and 2 h, respectively, Fr. F dose dependently, increased wound closure by 76 and 95% at 250 μg/mL, respectively (P < 0.01, after comparing with the control group) (Figs. 3 and 4). According to the results, Fr. F stimulated anti-inflammatory activity, cell proliferation and migration, indicating a wound-healing effect, and was the most active fraction in step I of fractionation, so it was selected for further fractionation in the next step.

### Step II: FF-SUB_1_ and FF-SUB_2_ were the most active subfractions from Fr. F

Fr. F was fractionated using column chromatography with silica gel, yielding five subfractions FF-SUB_1_ to FF-SUB_5_, which were investigated for their potential for wound healing.

#### In vitro anti-inflammatory study

**Figure 5:**
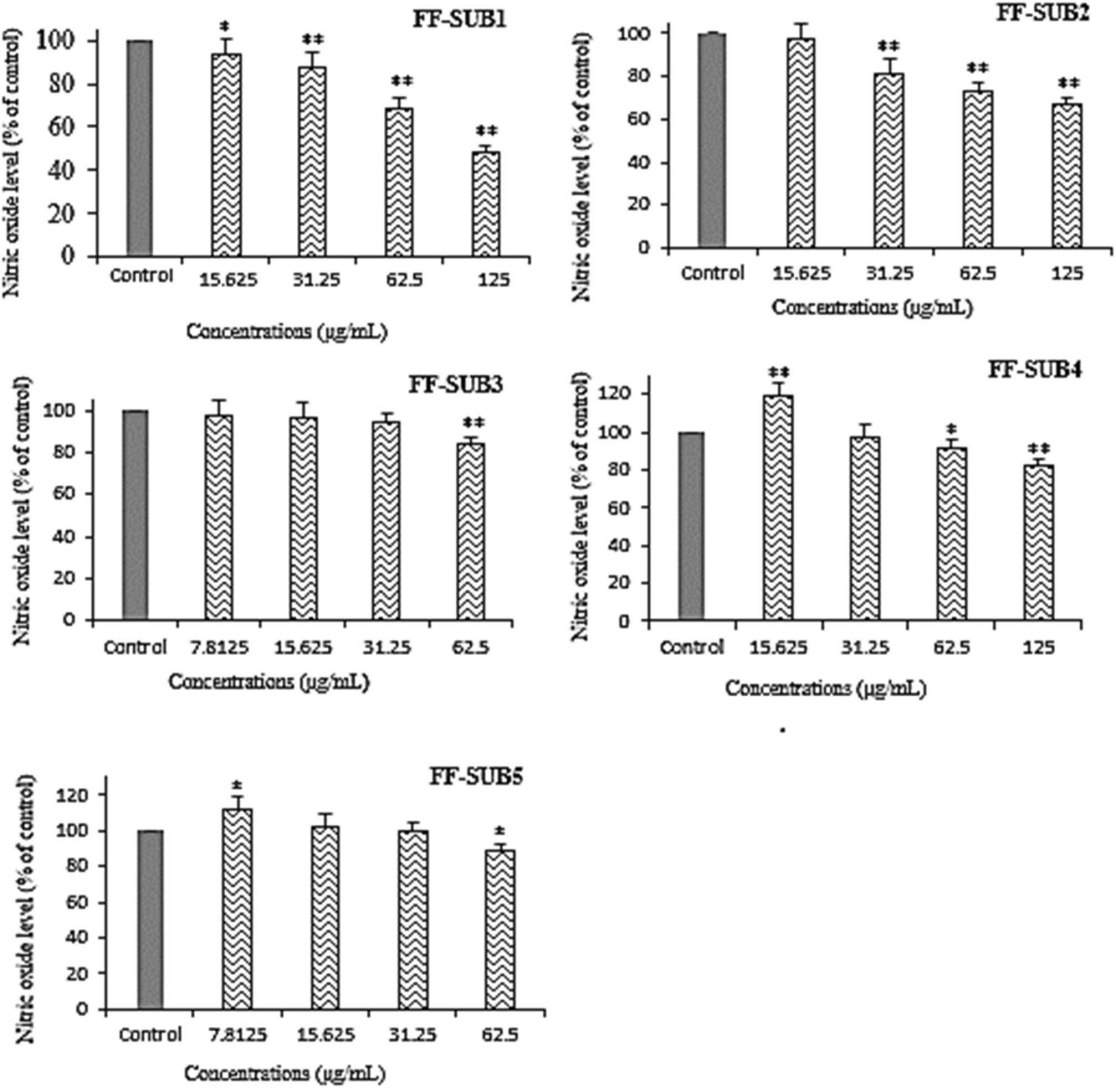
Effects of five subfractions of Fr. F, namely FF-SUB_1_ to FF-SUB_5_, on nitric oxide production by RAW 264.7 cells after treatment with LPS. All bars and values represent the mean ± SD of at least three replicate experiments. *Significant difference (P < 0.05) and **P < 0.01 when compared to the control group.

As shown in Fig. 5, FF-SUB_1_ and FF-SUB_2_ at concentrations of 125 µg/mL inhibited the production of nitric oxide in LPS-induced RAW264.7 cells by 51.5 ± 5.2% and 32.9 ± 3.4%, respectively with P < 0.01 when compared to the control group. The effect of FF-SUB_3_, FF-SUB_4_, and FF-SUB_5_ was 15.5 ± 3.5%, 18 ± 6.1%, and 11.2 ± 3.4%, respectively, at maximal nontoxic concentrations.

#### Fibroblast proliferation assay

**Figure 6:**
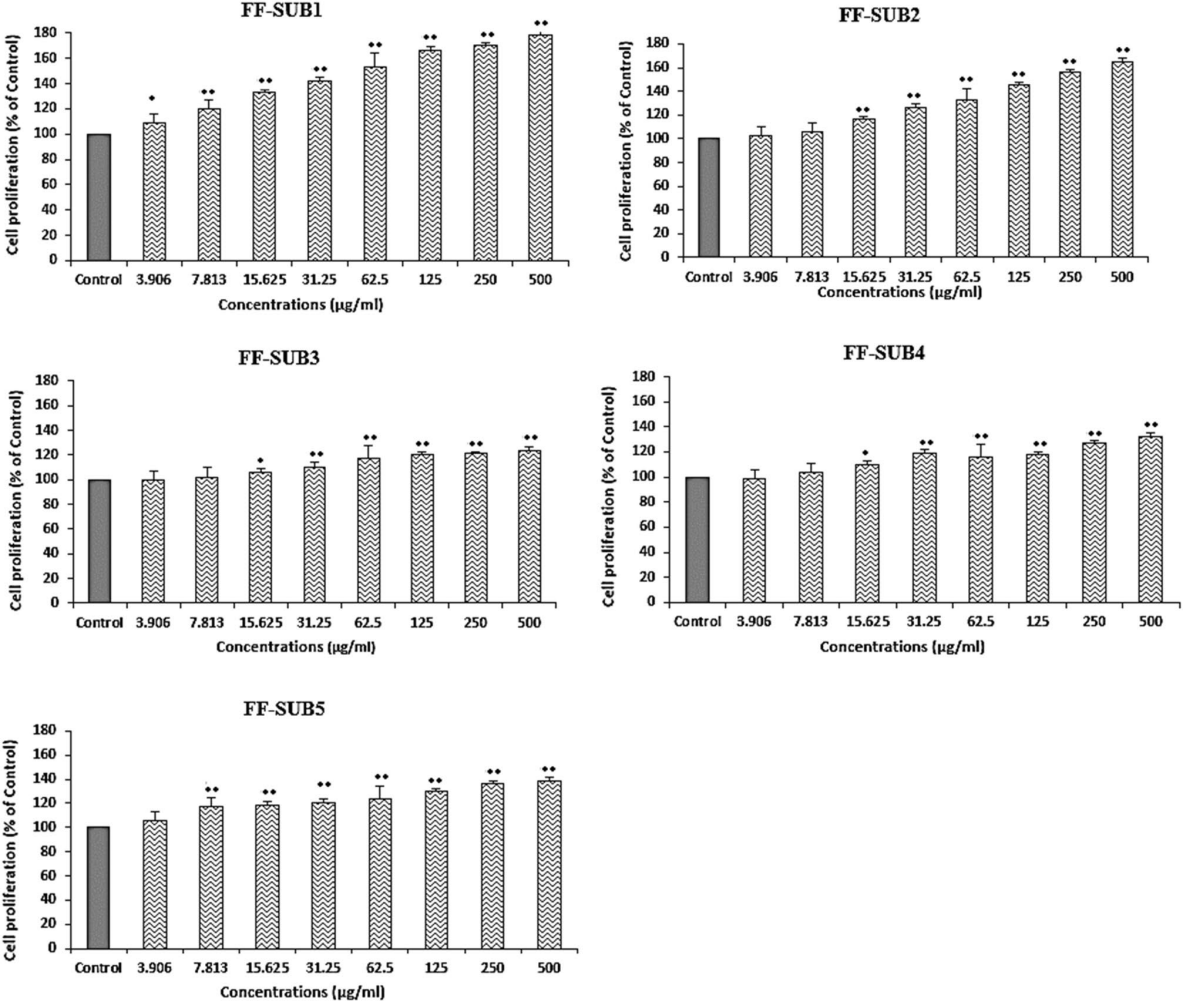
Effects of five subfractions of Fr. F, namely FF-SUB_1_ to FF-SUB_5_, on the proliferation rate of Hs.27 fibroblast cells. All bars and values represent the mean ± SD of three replicate experiments. *Significant difference (P < 0.05) and **P < 0.01 when compared to the control group.

As can be seen in Fig. 6, FF-SUB_1_ and FF-SUB_2_ showed the highest dose-dependent effect among the five subfractions, with growth of 78% and 65%, respectively, at a concentration of 500 μg/mL (P < 0.01 when compared to the control group). FF-SUB_3_, FF-SUB_4_, and FF-SUB_5_ induced growth of 23%, 31%, and 38%, respectively, in the HS.27 fibroblast cells at the highest concentrations used (500 μg/mL). As seen in Figs. 5 and 6, the wound healing effects of Fr. F were distributed mainly in two subfractions, namely FF-SUB1 and FF-SUB2, and an increase in anti-inflammatory and cell proliferative activity was observed from fractions to subfractions. Based on the present results, FF-SUB_1_ and FF-SUB_2_ were selected for purification.

#### Structure elucidation of the main compounds from FF-SUB_1_ and FF-SUB_2_

**Figure 7:**
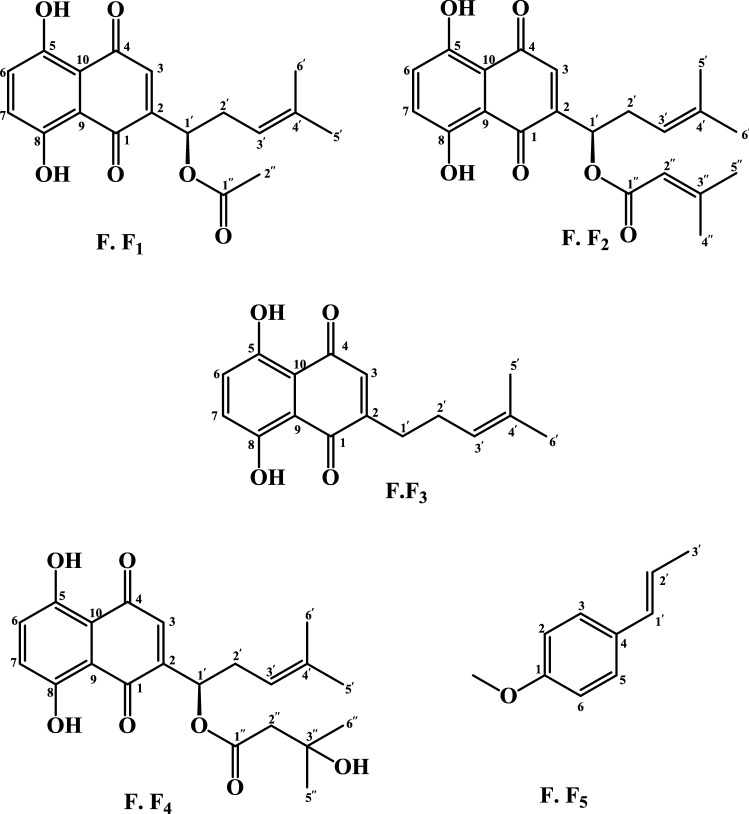
Chemical structure of purified compounds from the cyclohexane extract of O. *dichroanthum*. Roots.

The isolated compounds from the FF-SUB_1_ and FF-SUB_2_ subfractions were characterized based on NMR data and comparisons with the literature. Accordingly, four known naphthoquinones and one phenylpropanoid derivative were identified: Acetylshikonin (**F. F**_**1**_), β-β-dimethylacrylshikonin (**F. F**_**2**_), deoxyshikonin (**F. F**_**3**_), β-hydroxyisovalerylshikonin (**F. F**_**4**_), and trans-anethole (**F. F**_**5**_) (Fig. 7).

- **F.F**_**1**_^1^H-NMR (CDCl_3_, 400 MHz) δ: 12.66 (phenolic OH, *s*, 1H), 12.5 (phenolic OH, *s*, 1H), 7.34 (H-6 and H-7, *s*, 2H), 7.06 (H-3, *d*, *J* = 0.8 Hz, 1H), 6.07 (H-1′, *m*, 1H), 5.19 (H-3′, *t*, *J* = 7.2 Hz, 1H), 2.56–2.69 (H-2′, *m*, 2H), 2.3 (H-2′′, *s*, 3H), 1.77 (H-H-5′, *s*, 3H), 1.65 (H-6′, *s*, 3H), ^13^CNMR (CDCl_3_, 125 MHz) δ: (C-4, 178.3), (C-1, 176.8), (C-1′′, 169.9), (C-8, 167.6), (C-5, 167.1), (C-2, 148.4), (C-4′, 136.3), (C-6, 133.0), (C-7, 132.9), (C-3, 131.6), (C-3′, 117.7), (C-9, 112.01), (C-10, 111.7), (C-1′, 69.7), (C-2′, 33.02), (C-5′, 25.9), (C-C-2′′, 21.1), (C-6′, 18.1).

Compound F.F_1_ was identified as shikonin acetate (acetylshikonin) by comparing its spectroscopic data with literature data^21^.

- **F.F**_**2**_ The ^1^H NMR (CDCl_3_, 400 MHz) of this compound agrees with the literature data^22^ and compound F.F_2_ was identified as β, β-dimethylacryl shikonin.
- **F.F**_**3**_ The ^1^H NMR (CDCl_3_, 400 MHz) of this compound is agreeing with the literature data^21,23,24^, so compound F.F_3_ was identified as deoxyshikonin.
- **F.F**_**4**_ The ^1^H NMR (CDCl_3_, 400 MHz) of this compound is in agreement with the data^25,26^ reported in the literature, so compound F.F_4_ was identified as β-hydroxyisovalerylshikonin.
- **F.F**_**5**_ The ^1^H NMR (CDCl_3_, 400 MHz) of this compound is in agreement with the data^27^ in the literature, so compound F.F_5_ was identified as trans-anethole.

Naphthoquinone compounds have been reported in almost all studies on Onosma species, but this is the first report of the presence of trans-anethole in a species of this genus.

## Discussion

The wound-healing effect of cyclohexane extract of *O. dichroantha* has been previously demonstrated by assays of anti-inflammation, cell proliferation, cell migration, and angiogenesis^20^. Therefore, we conducted this study in order to fractionate and purify active constituents from cyclohexane extract using bioassay methods. However, some limitation should be noted. The first issue was time constraints. As the scratch test takes a long time to form a uniform layer of HMEC-1 cells, it was not evaluated for the subfractions in this study, so only the two remaining wound healing tests were performed. Additionally, there was no access to the most widely used methods for measuring anti-inflammatory properties, which rely mostly on gene expression levels or directly measuring DNA synthesis to assess cell proliferation. Therefore, three convenient and reliable cell culture experiments were used for quick screening and comparison.

Bacterial LPS can stimulate the release of a variety of inflammatory mediators, such as nitric oxide, in different cell types. Therefore, it can be used to evaluate anti-inflammatory activities in vitro. Nitric oxide as a free radical, when produced in excess, can directly damage the function of normal cells and cause inflammation. This is because it binds to superoxide radicals and forms peroxynitrite, which is an unstable isomer of nitrate and highly reactive^28,29^. The NO concentration in the culture medium was measured colorimetrically using the Griess reaction. Comparison of Figs. 1 and 5 show that the strongest inhibition of nitric oxide production was observed with FF-SUB1, followed by Fr. F and FF-SUB2.

The proliferative phase of fibroblasts, which play a crucial role in repair processes, is very important for tissue regeneration in wound healing. According to Fig. 2, Fr. F at a concentration of 500 µg/mL had the strongest effect on fibroblast cell proliferation. However, after using a higher concentration of Fr. F, signs of cytotoxicity were observed. Since this cytotoxicity occurred at higher doses, precautions should be taken when using this plant as a wound healing agent.

The multicellular structure of cells during cell migration gives them the ability to respond to physical, chemical, and biological signals compared to unicellular cells. This advantage is crucial because epithelial cells can migrate and heal wounds as a large, interconnected group of cells with tight junctions^30^. In our study, we determined the effect of *O. dichroantha* fractions on the cell migration of human microvascular endothelial cells (HMEC-1) as measured by the scratch wound assay. This method is commonly used as an in vitro technique to evaluate cell migration ability. It has been used in many studies to date as an indicator of the cell migration rate induced by plant extracts or purified compounds of these extracts^31–33^. Scratches are created by creating an artificial gap in a cell monolayer along the tip of a pipette^34^.

Further purification of the active subfractions eventually led to the purification of compounds with naphthoquinone structures and a phenylpropanoid derivative called trans-anethole. The presence of most of the active components isolated in this study has been previously reported in other species of the genus Onosma^22–24,26^. However, our study is the first to describe the presence of trans-anethole in a species of this genus. Naphthoquinone compounds have been identified, isolated, and purified in almost all wound healing studies conducted on the roots of the genus Onosma. The wound healing activity of these compounds has been investigated and described in previous literature.

Four naphthoquinone compounds from the dichloromethane-hexane extract of *O. argentatum* were purified^23^ and the stimulation of fibroblast proliferation by these compounds as a critical factor in promoting wound healing activity using human fibroblasts in the in vitro condition was investigated. Since both the hexane dichloromethane extract (1:1) and the compound 5, 8-O-dimethylacetylchikonin stimulated the proliferation of fibroblast cells, their results confirm the traditional use of this plant for wound healing^35^. The efficacy of β, β-dimethylacrylshikonin on HUVEC cell migration in a scratch assay and the pre-angiogenic effect on tube formation was evaluated^36^. This compound, in combination with a small amount of VEGF, showed a synergistic effect. It improved cell proliferation and migration and wound closure rate in diabetic wounds induced in rats. The anti-inflammatory properties of β-hydroxyisovalerylshikonin (β-HIVS) were investigated in several studies: in a study, the production of NO and prostaglandin E2 in the presence and absence of (β-HIVS) in BV-2 cells was investigated^37^. In cells pre-treated with β-HIVS, the production of NO as well as of PGE2 decreased in a dose-dependent manner. At the molecular level, the expression of the LPS-induced genes iNOS and cyclooxygenase-2 was significantly inhibited after the treatment of cells with β-HIVS. The high efficacy of β-HIVS in reducing inflammation in osteoarthritis was demonstrated by decreasing the expression of PEG2, NO, iNOS, interleukin (IL)-6, (TNF)-α, and COX-2 in chondrocyte cells^38^. Comparison of wound healing efficiency between acetylshikonin and gentamicin and silver sulfadiazine ointments after 7 days of application in several groups of Sprague-Dawley rats with wounds that were both sterilized and infected with Pseudomonas aeruginosa and Staphylococcus aureus showed that the rate of new epithelial formation in all groups of rats was significantly higher in the presence of acetylshikonin than with the ointments used. Histologic studies confirmed the significant formation of granular tissue, angiogenesis, and formation of new epithelia in all acetylshikonin-treated rats^39^. One study showed that treatment of HUVECs cells with 3 μM as the most effective concentration of deoxyshikonin increased tube formation in the angiogenesis assay. Cell adhesion also increased by 80% at the same deoxyshikonin concentration. Other results of this study included cell proliferation induced by this agent in HaCaT cells and the strongest wound closure in a mouse model at 20 Μm^40^. In a continuation of previous studies, the effect of deoxyshikonin on the activation of the kinase function of the ERK/MAPK pathway and p38 through phosphorylation was investigated. ERK/MAPK is one of the majors signaling pathways involved in tube formation and cell proliferation.

The results showed a 1.42-fold and 2.97-fold increase in gene expression for p-ERK and p-p38, respectively, compared with the control group^41^.

The topical wound poses a high risk of bacterial, fungal, and viral infection, so it must be treated with special care. Pathogens employ a number of mechanisms to impede the healing process. Medicinal plants are effective in treating infectious disorders and infections of many types of external wounds^42–44^. The antibacterial activity of the ethanolic root extract of *O. dichroantha* was investigated by Pahlavan et al.^45^ by utilizing the disk diffusion method. The results showed that the extract had high bioactivity at 100 mg/mL, with zones of inhibition ranging from 6.0 ± 0.00 to 16.3 ± 1.52 mm on *Staphylococcus aureus, Pseudomonas aeruginosa, Streptococcus pneumoniae, Streptococcus agalactiae, Enterococcus faecalis, Escherichia coli, Shigella sonnie, Shigella flexteriae, Listeria monocytogenes, Staphylococcus epidermis* and *Streptococcus pyogenes*. In comparison with the standard controls, the inhibition value was significant.

In general, shikonin and its derivatives have been shown to have a wide range of properties, including antioxidant, anti-inflammatory, neuroprotective, cardioprotective, anticancer, antimicrobial, and wound healing properties^46–52^.

Several naphthoquinone compounds, including deoxyshikonin, 5, 8-O-dimethyl isobutyrylshikonin, isobutyrylshikonin, acetylshikonin, β-hydroxyisovalerylshikonin, α-methylbutyrylshikonin, and 5, 8-O-dimethyl deoxyshikonin were isolated from *O. visanii* roots. These compounds demonstrated significant antimicrobial activity against gram positive and gram-negative bacteria, with MIC50 and MIC90 values ranging from 4.27 to 68.27 g/mL and 4.77–76.20 g/mL, respectively^53^.

In addition to being a naturally occurring biologically active compound, trans-anethole has been shown to have a wide range of beneficial effects on human health, including anti-inflammatory, neuroprotective, anticoagulant, antihypertensive, anticancer, antibacterial, immunoregulatory, and antidiabetic effects. Trans-anethole may be effective in treating various chronic diseases, including inflammatory diseases of the skin and lungs, cancer, type 2 diabetes, and neurological diseases. It appears that the mechanisms being studied to demonstrate the efficacy of trans-anethole are related to the modulation of several signaling pathways, particularly the MAPK, TNF-α, and NF-kB pathways. Clearly, long-term clinical studies are needed to confirm its validity. In this context, studies showed that trans-anethole reduced inflammation by inhibiting cellular responses to TNF and inhibiting the release of mediators, such as nitric oxide^54^.

In a study, a topical formulation of essential oils from *Croton zehntneri* leaves was prepared in Pluronic-127 (2% and 20% essential oils). 85.7% of the essential oils in the leaves of this plant are trans-anethole. Then, wounds were created in full thickness on the left and right back of the mice. This topical formulation was applied to mice twice daily for 15 days. The effect of topical application of trans-anethole on wound tissue repair was studied and compared with dexamethasone as a control group. Three days after the start of wound treatment, it was found that treating the wounds with a formulation containing 20% essential oil and dexamethasone in equal amounts reduced wound swelling and drainage. In mice treated with both sets of formulations, the wound closure rate and the increase in the number of fibroblast cells and collagen fibers were observed^55^. The antimicrobial activities of trans-anethole-rich oil derived from some varieties of fennel were assessed using the broth dilution method to determine the minimum inhibitory concentration (MIC) and minimum bactericidal concentration (MBC). In this study, eight gram-positive and gram-negative bacteria species were used. The results show a significant effect, particularly on gram-positive bacteria, with MIC and MBC values ranging from 25 to 100 μg/mL^56^.

## Conclusions

Overall, the present study shows that the fractions and subfractions of cyclohexane extract of *O. dichroantha* roots mainly contain naphthoquinone derivatives, which have remarkable anti-inflammatory, cell proliferation and cell migration-promoting properties when bioassay-guided fractionation methods are used. Thus, they have the potential to be an effective wound healing agent. It seems that further studies should be conducted to find out the clinical doses of these fractions and subfractions, as well as the purified compounds, using in vivo models.

## Materials and methods

### Plant material

In the autumn, at the end of the first year of a plant's life cycle, we collected roots from this plant in the Hezar Jerib region of Mazandaran province at an altitude of 1800 m above sea level (as indicated by WHO guidelines on good agricultural and collection practices for medicinal plants (GACP), 2003)^57^. Plant authentication was performed by an expert botanist (Joharchi, Mohammad Reza) at the Institute of Plant Science, University of Ferdowsi, Mashhad, Iran. A voucher specimen (36,194) was deposited in the herbarium. This common, widespread plant is not an endangered plant, and the collection of samples with the permission of the university was done only for academic study by observing the necessary guidelines for collecting plants (IUCN Policy Statement on Research Involving Species at Risk of Extinction and the Convention on the Trade in Endangered Species of Wild Fauna and Flora).

### Bioassay-guided fractionation of cyclohexane extract

Based on our previous data^20^, the cyclohexane extract was selected for further experiments by bioassay-guided fractionation. This fractionation was performed in two steps, with three in virto assays following each step. At step I, 15 g of the CE extract were fractionated using column chromatography with silica gel (230–400 mesh, Merck KGaA, Darmstadt, Germany, 120 cm × 10 cm) and eluted with a gradient of *n*-hexane-dichloromethane (90:10, 50:50, 30:70), ethyl acetate (100), acetone (100), and finally acetone–water (80:20) to yield 6 fractions (Fr. A, 1.36 g; Fr. B, 2.65 gr; Fr. C, 0.71 g; Fr. D, 0.75 g; Fr. E, 3.18 g; Fr. F, 5.40 g), which each group had similar phytochemical constituents in thin layer chromatography. We selected the fraction/s with the most biological activity in all three mechanisms of the wound healing process for further fractionation in step II. Based on the biological activities (see results later), Fr. F (5.40 g) was fractionated using column chromatography on the same silica gel (230–400 mesh, 120 cm × 2.5 cm) and eluted successively with chloroform–methanol (95:5, 90:10, 85:15) to obtain five subfractions: FF-SUB_1_ (2.36 g), FF-SUB_2_ (0.88 g), FF-SUB_3_ (0.534 g), FF-SUB_4_ (0.458 g) and FF-SUB_5_ (0.761 g). Again, based on the biological activities shown in the later results, FF-SUB_1_ and FF-SUB_2_ were selected for further fractionation and purification.

FF-SUB_1_ (2.36 g) was then applied to column chromatography (15 cm × 2 cm) on silica gel and eluted successively with chloroform-cyclohexane (70:30, 80:20, and 90:10) to give five subtractions (FF-A_1_ to FF-A_5_). FF-A_1_ was subjected to column chromatography (15 cm × 1.5 cm) on Sephadex LH-20 (Amersham Biosciences, UK), eluted with chloroform–methanol (50:50), followed by preparative reversed-phase TLC, eluted with acetonitrile to give compound F. F_2_ (53 mg) as a reddish crystal substance with dark red color. FF-A_2_ was subjected to the same column chromatography on Sephadex LH-20 and then eluted by preparative reversed-phase TLC with acetonitrile: butanol: water (80:20:2) to give F. F_1_ (71 mg) as a red solid substance. Successively, Sephadex LH-20 columns were applied in the same manner to FF-A_4_, resulting in two subfractions (FF-A_4_-B_1_ and FF-A_4_-B_2_). FF-A_4_-B_1_ was developed by preparative reversed-phase TLC using petroleum ether-CHCl_3_-MeOH water (20:60:3:2) to give F. F_3_ (36 mg) as a red oily substance. F. F_4_ (26 mg) as a red powder was purified from FF-A_4_-B_2_ by preparative reversed-phase TLC eluted with acetonitrile:butanol:water (80:20:2). FF-SUB_2_ (0.88 g) was subjected to column chromatography (15 cm × 2 cm) on silica gel and eluted successively with chloroform-cyclohexane (70:30, 80:20, 90:10, and 100:0) to give six minor subfractions (FF-A'_1_ to FF-A'_6_). The major subfraction, FF-A'_1_, was separated by preparative silica gel TLC and eluted with chloroform-cyclohexane (50:50) to afford compound F. F_5_ as a yellow oil (46 mg).

### Structure elucidation of the pure compounds (F. F_1_–F. F_5_)

The structures of the purified compounds were elucidated using NMR (nuclear magnetic resonance) spectrometry (Bruker Avance-400 NMR spectrometers).

### Cell culture

The mouse monocyte/macrophage cell line (RAW 264.7 cells), normal human skin fibroblast cells Hs.27, and HMEC-1, a human dermal microvascular endothelial cell line, were purchased from the American Type Culture Collection (ATCC, Virginia, USA). RAW264.7 cells and Hs.27 fibroblasts were cultured in DMEM (Dulbecco's Modified Eagle's Medium) containing high glucose, 10% (v/v) FBS (fetal bovine serum), and 1% (v/v) PS (penicillin–streptomycin) (Invitrogen Corporation, CA, USA). HMEC-1 cells were cultured in MCDB 131 media containing 10 ng/mL EGF (human epidermal growth factor), hydrocortisone (1 μg/mL), and 10% FBS. All cells grew in monolayers in humidified incubators at 5% CO_2_ and 37 °C. All in vitro methods used were in accordance with those described elsewhere^31^.

### Cell viability

The MTT assay (3–4,5 dimethylthiazol-2-yl-2,5-diphenyltetrazolium bromide) was used to measure the viability of HMEC-1 cells and mouse macrophages RAW 264.7. RAW 264.7 cells and HMEC-1 cells were seeded at a rate of 10^5^ cells per well on 96-well plates. Plates were then incubated for 24 and 48 h, respectively. Stock solutions of six fractions and five subfractions were prepared in DMSO. Working solutions were prepared at concentrations from 250 to 1.953 μg/mL for RAW 264.7 and HMEC-1 cells by diluting the respective volumes of stock solutions with culture medium and using them to treat the cells. The cells were further cultured with the prepared samples and placed in the incubator for 24 or 48 h. Then MTT solution (30 μL, 0.5 mg/mL) was added to each well and incubated for a further 4 h. Formazan crystal formation was detected after 10 min at room temperature by evaluating the optical density at 570 nm. The experiment was repeated four times.

### Anti-inflammatory assay

LPS (lipopolysaccharide) was used to induce inflammation in cells. To investigate the anti-inflammatory properties, RAW264.7 cells were seeded in a 24-well plate with 4 × 10^5^ cells per well and incubated for 24 h. Then, the medium was replaced with LPS solution (500 μL, 0.2 μg/mL) and the same volume of fractions and subfractions at their nontoxic concentrations ranging from 31.25 to 3.91 μg/mL of Fr. A, 3.91–0.488 μg/mL of Fr. B, Fr. C, Fr. D, 15.625–1.95 μg/mL of Fr. E, 125–15.625 μg/mL of Fr. F and 125–15.625 μg/mL of FF-SUB_1_, FF-SUB_2_ and FF-SUB_4_, 62.5–7.81 μg/mL of FF-SUB_3_ and FF-SUB_5_, respectively. The plates were again placed in the incubator for 24 h. Measurement of nitrite concentration in the supernatant, an inert and stable metabolite of nitric oxide, was considered as an index of NO production using the nitrite assay kit (Griess reagent (modified), Sigma-Aldrich). Briefly, two 50 μL culture supernatants are taken from each well and transferred to adjacent wells of 96-well plates. After adding an equal volume of Griess reagent to each well, the plates are stored in the dark at room temperature for 15 min. The absorbance was then measured at 540 nm using an ELISA reader. A standard curve for sodium nitrite was plotted and the nitrite concentration in the samples treated with fractions and subfractions was determined from the curve. The experiment was repeated three times.

### Cell proliferation assay

The MTT assay was used as an indicator of cell proliferation to evaluate the efficacy of fractions and subfractions on cell proliferation. Hs.27 Fibroblast cells (3 × 10^3^ cells per well, 96-well plate) were incubated for 24 h. Then, the medium was aspirated and 200 μL of different concentrations of samples (from 1000 to 7.81 μg/mL of fractions and from 500 to 3.91 μg/mL of subfractions) were added. The plates were placed in the incubator for 48 h. Then, MTT solution (30 μL, 0.5 mg/mL) was added to each well and incubated for an additional 4 h. Finally, all medium in each well was replaced with 100 μL DMSO. The absorbance was then measured at 540 nm using an ELISA reader. The experiments were performed at least three times.

### Cell migration assay

To study cell migration and wound healing, the "in vitro scratch assay" method was performed. HMEC-1 cells were seeded in a 24-well plate with 10^5^ cells per well and incubated for 24 h. After this time, all medium was withdrawn and all cells were allowed to starve in culture medium containing 1% FBS and 1% PS for 24 h. Then, two vertical scratches in the shape of a cross were made in each well with a pipette tip (100 μL) and photographed as time 0 with a fluorescence microscope with camera (Nikon Eclipse TS100) with × 4 magnification. All medium was withdrawn and replaced with nontoxic concentrations of Fr A (from 31.25 to 1.95 µg/mL), Fr B and Fr D (from 1.95 to 0.06 µg/mL), Fr C (from 3.91 to 0.12 µg/mL), Fr E (from 31.25 to 0.977 µg/mL), and Fr F (from 250 to 7.81 µg/mL). Fresh medium served as a control. Plates were further incubated, and images were recaptured at the same locations after 16 and 24 h. Tscratch software was used to analyze the images^58^. The percentage increase in wound closure compared to the value obtained before treatment was measured and reported as cell migration. This test was performed at least three times.

### Statistical analysis

All values obtained within groups were presented as mean ± standard deviation. SPSS version 22.0 (IBM Corp., USA) was used for statistical analyses. One-way ANOVA (analysis of variance) followed by the Tukey test was performed to determine statistical significance. P < 0.05 was considered statistically significant. To compare the relationship between each concentration at 16 and 24 h, the unpaired samples t-test was used.

## Supplementary Information

Supplementary Information.

## Data Availability

All data generated or analyzed during this study are included in this published article [and its Supplementary information files].
