# Supplementary material for: In vitro wound healing potential of cyclohexane extract of Onosma dichroantha Boiss. based on bioassay-guided fractionation
Source: Sci Rep. 2023 Mar 28;13:5018. doi: 10.1038/s41598-023-31855-7 (PMC10050168; doi:10.1038/s41598-023-31855-7)
Supplement: Supplementary file 1 — Supplementary Information. [file 41598_2023_31855_MOESM1_ESM.pdf]

SUPPLEMENTARY MATERIAL

In vitro wound healing potential of cyclohexane extract of *Onosma dichroantha* Boiss. base on bioassay-guided fractionation

Fereshteh Safavi [1], Mahdi Moridi Farimani [2*], Masoud Golalipour [3], Houman Bayat[4]

[1] Department of Chemistry, Faculty of Science, Golestan University, Gorgan, Iran

[2] Department of Phytochemistry, Medicinal Plants and Drug Research Institute, Shahid Beheshti University, Evin, Tehran 1983969411, Iran

[3] Medical Cellular and Molecular Research Center, Golestan University of Medical Sciences, Gorgan, Iran

[4] Niak Pharmaceutical Company, Golestan, Gorgan, Iran

CONTACT

Tel: +982129904043. Fax: +982122431783. E-mail: m_moridi@sbu.ac.ir.

List of Contents

Table S1. ^{1}H NMR Spectral Data of compounds **F.F$_{2-5}$** (δ_H in ppm, *J* in *HZ*)

Figure S1. A flow chart showing the bioassay-guided fractionation procedure of *O. dichroantha* cyclohexane extract.

Figure S2. ^{1}H NMR spectrum of compound **F.F$_1$ (**$CDCl_3$, 400 MHz**)**

Figure S3. ^{13}C NMR spectrum of compound **F.F$_1$** ($CDCl_3$, 125 MHz)

Figure S4. ^{1}H NMR spectrum of compound **F.F$_2$ (**$CDCl_3$, 400 MHz**)**

Figure S5. ^{1}H NMR spectrum of compound **F.F$_3$ (**$CDCl_3$, 400 MHz**)**

Figure S6. ^{1}H NMR spectrum of compound **F.F$_4$ (**$CDCl_3$, 400 MHz**)**

Figure S7. ^{1}H NMR spectrum of compound **F.F$_5$ (**$CDCl_3$, 400 MHz**)**

Table S1. ^{1}H NMR Spectral Data of compounds **F.F2-5** (δ_H in ppm, *J* in *HZ*)

Crbon No	Compounds (δ_H)			
	**F.F2**	**F.F3**	**F.F4**	**F.F5**
1	-	-	-	-
2	-	-	-	H 2 & 6: 7.27 (*d*, *J*=8.8 Hz, 2H)
3	7.06 (*s*, 1H)	6.85 (*s*, 1H)	7.10 (*s*, 1H)	H 3 & 5: 6.85 (*d, J*=8.8 Hz, 2H
**4**	-	-	-	-
**5**	-	-	-	-
**6**	H 6 & 7: 7.36 (*s*, 2H)	H 6 & 7: 7.21 (*s*, 2H)	H 6 & 7: 7.33 (*s*, 2H)	-
**7**				-
**8**	-	-	-	-
**9**	-	-	-	-
**10**	-	-	-	-
**1′**	6.10 (*ddd*, *J*=7.2, 4.5, 1.8 Hz, 1H)	2.63 (*t*, *J*=7.6 Hz, 2H)	6.17 (*m*, 1H)	6.38 (*d*, *J*=15.6 Hz, 1H)
**2′**	2.56- 2.71 (2*m*, 2H)	2.29 (*q*, *J*=7.6 Hz, 2H)	2.54 (*m*, 1H)<br>2.68 (*m*, 1H)	6.12 (*dq*, *J*=15.6, 6.8 Hz, 1H)
3′	5.24 (*t*, *J*=7.2 Hz,1H)	5.14 (*t*, *J*=7.1 Hz, 1H)	5.19 (*t*, *J*=7.6 Hz, 1H)	1.87 (*dd*, *J*=1.6, 6.8 Hz, 3H)
4′	-	-	-	-
5′	1.66 (*s*, 3H)	1.60 (*s*, 3H)	1.76 (*s*, 3H)	-
6′	1.77 (*s*, 3H)	1.70 (*s*, 3H)	1.66 (*s*, 3H)	-
**1″**	-	-	-	-
2″	-	-	2.65 (*d*, 2H)	-
3″	-	-		-
4″	2.24 (*s*, 3H)	-	1.38 (*s*, 3H)	-
5″	2.03 (*s*, 3H)	-	1.48 (*s*, 3H)	-
6″	-	-	-	-
-	12.51 (OH, *s*, 1H)<br>12.68 (OH, *s*, 1H)	12.50 (OH, *s*, 1H)<br>12.67 (OH, *s*, 1H)	3.25 (OH, *s*, 1H)<br>12.50 (OH, s, 1H)<br>12.70 (OH, s, 1H)	3.82 (OCH_3, *s*, 3H),

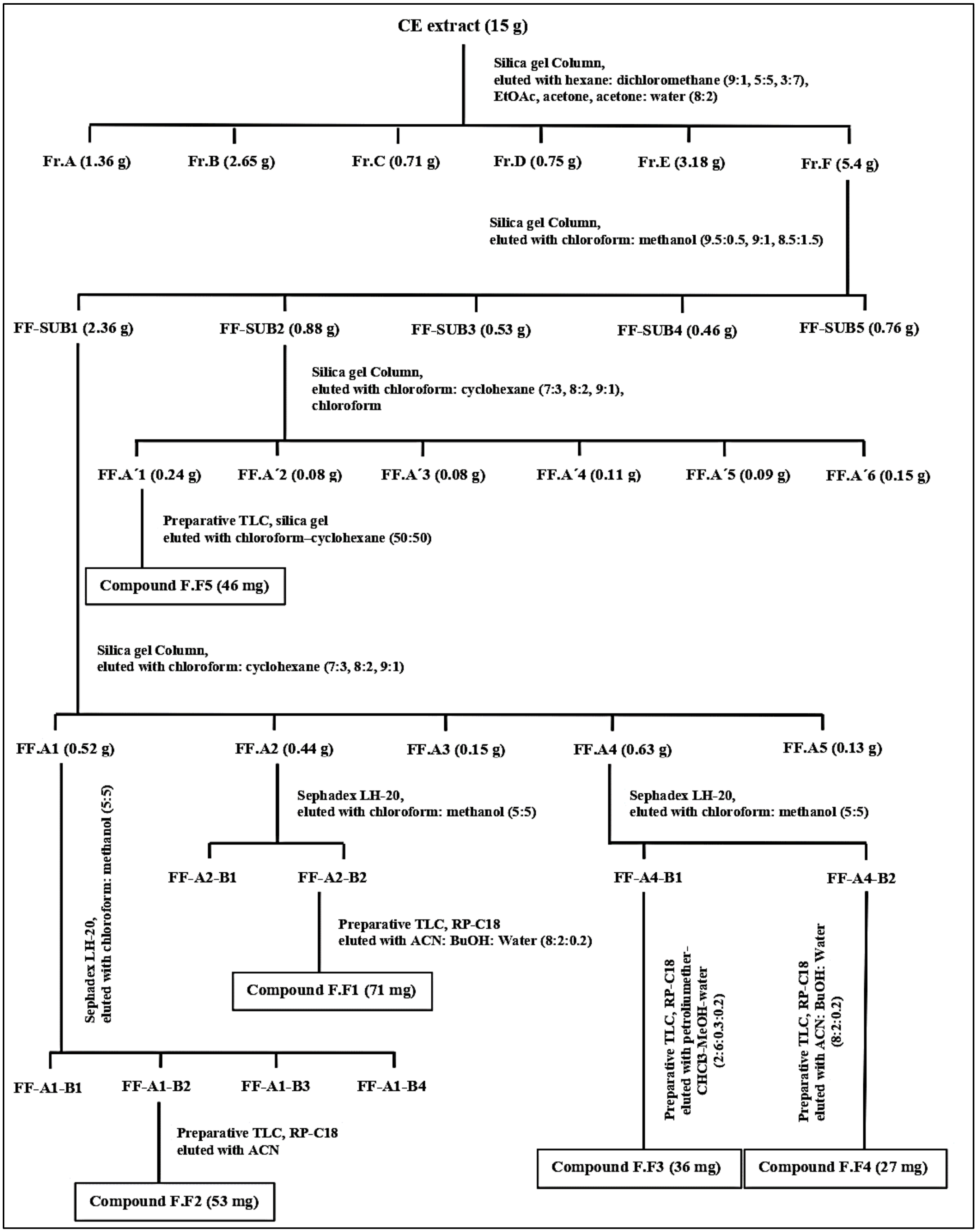

Fig. S1. A flow chart showing the bioassay-guided fractionation procedure of *O. dichroantha* cyclohexane extract.

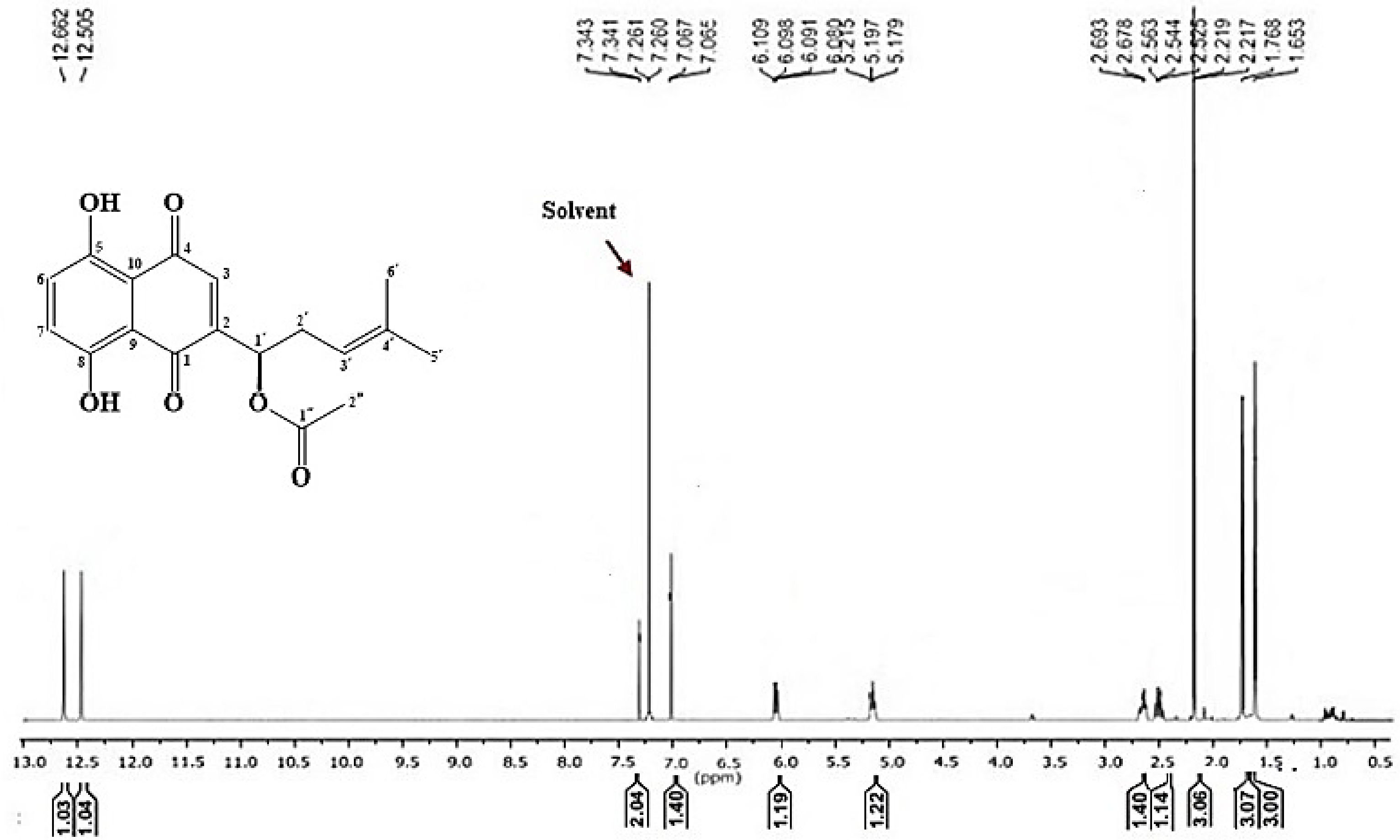

Figure S2. ^{1}H NMR spectrum of compound **F.F$_1$ (**$CDCl_3$, 400 MHz**)**

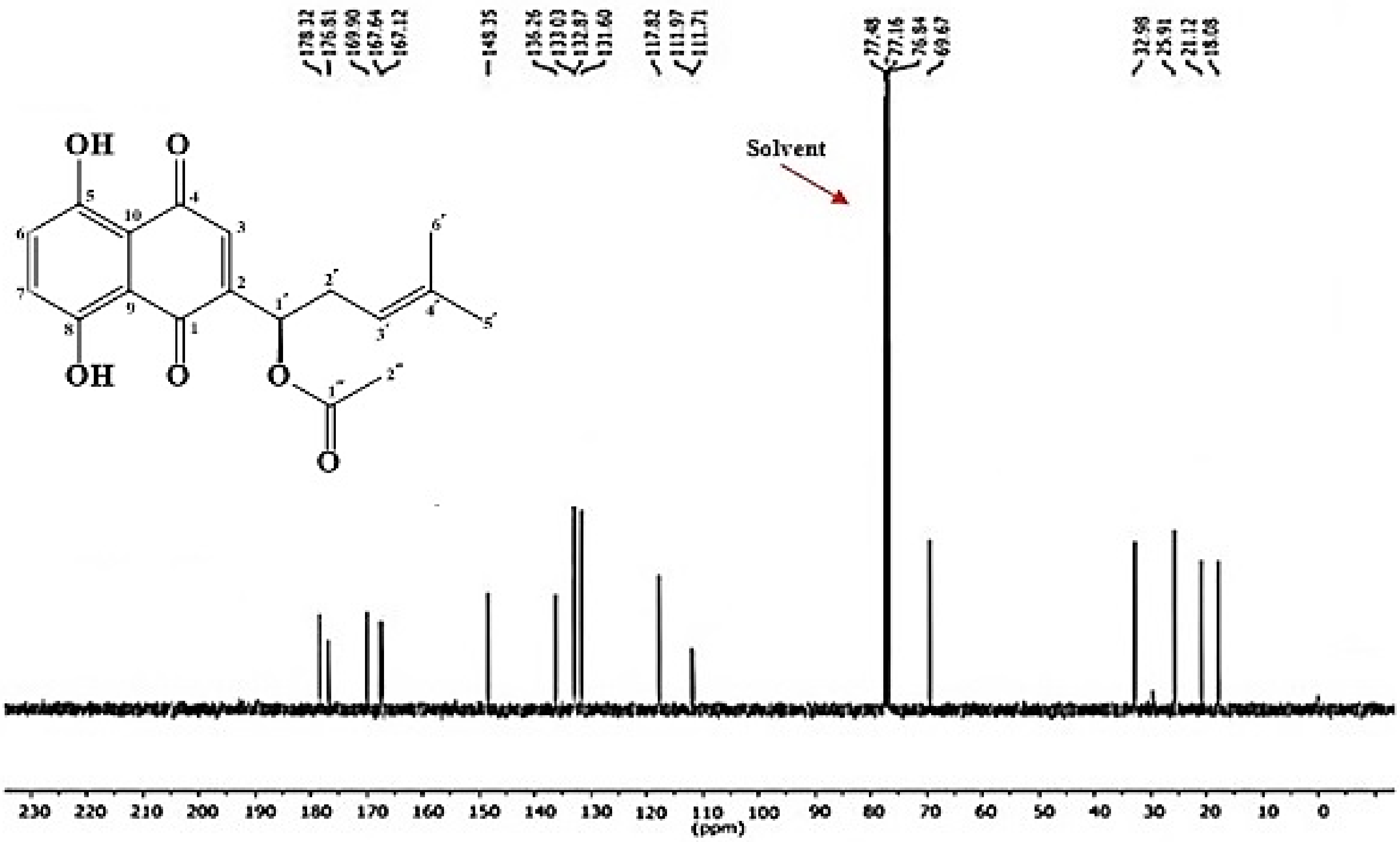

Figure S3. ^{13}C NMR spectrum of compound **F.F$_1$** ($CDCl_3$, 125 MHz)

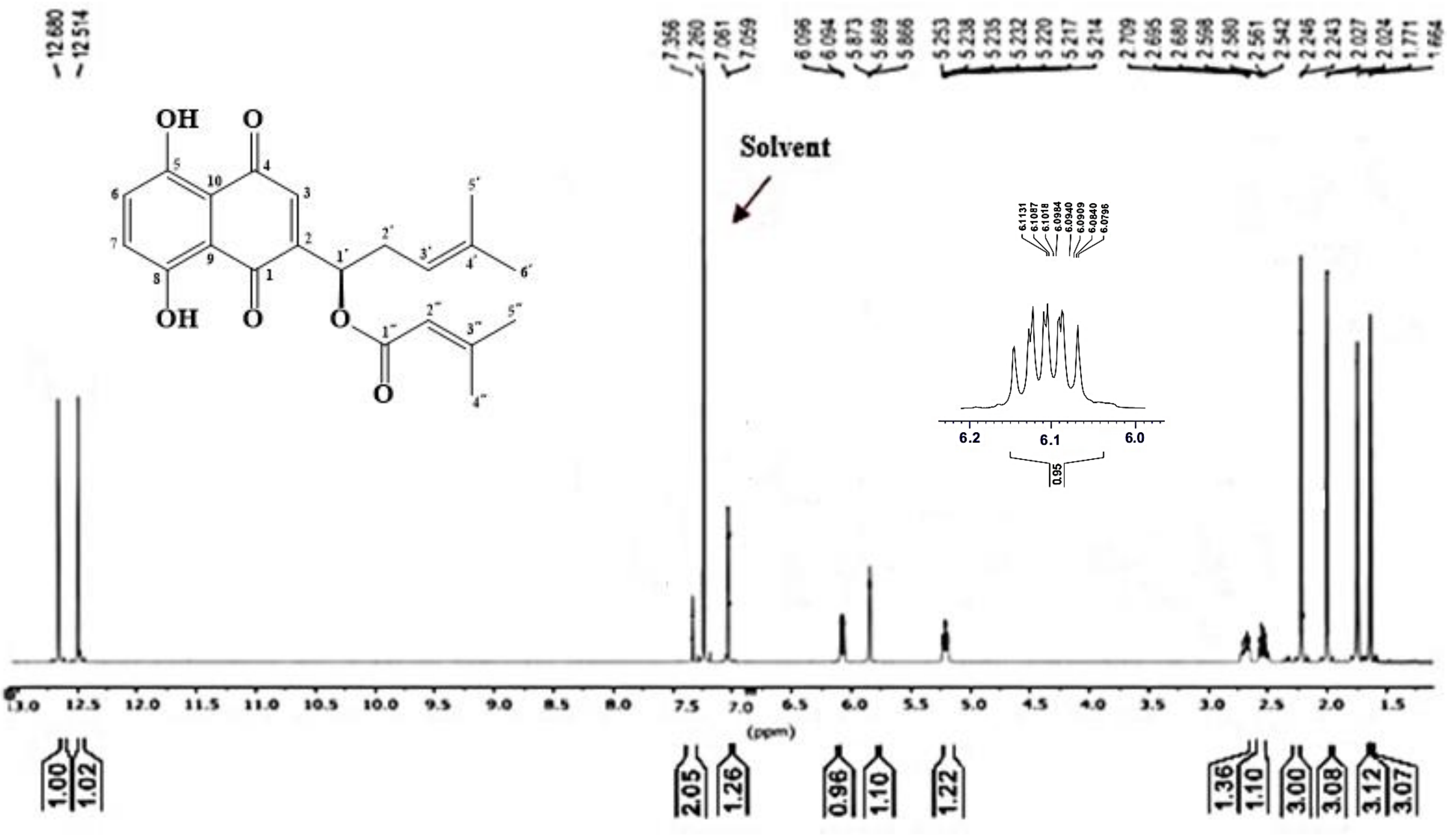

Figure S4. ^{1}H NMR spectrum of compound **F.F$_2$** (CDCl$_3$, 400 MHz

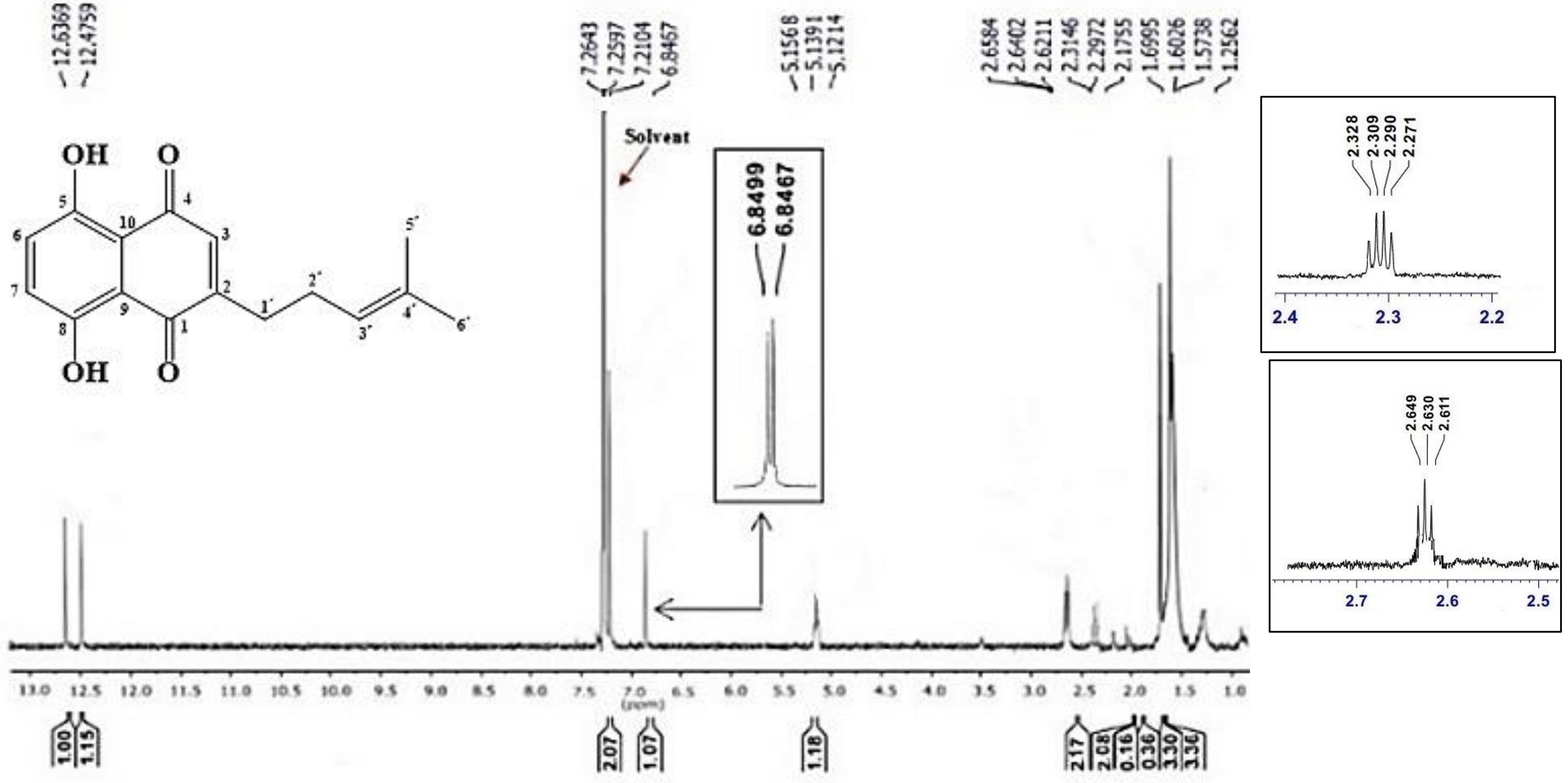

Figure S5. ^{1}H NMR spectrum of compound **F.F3 (**$CDCl_3$, 400 MHz**)**

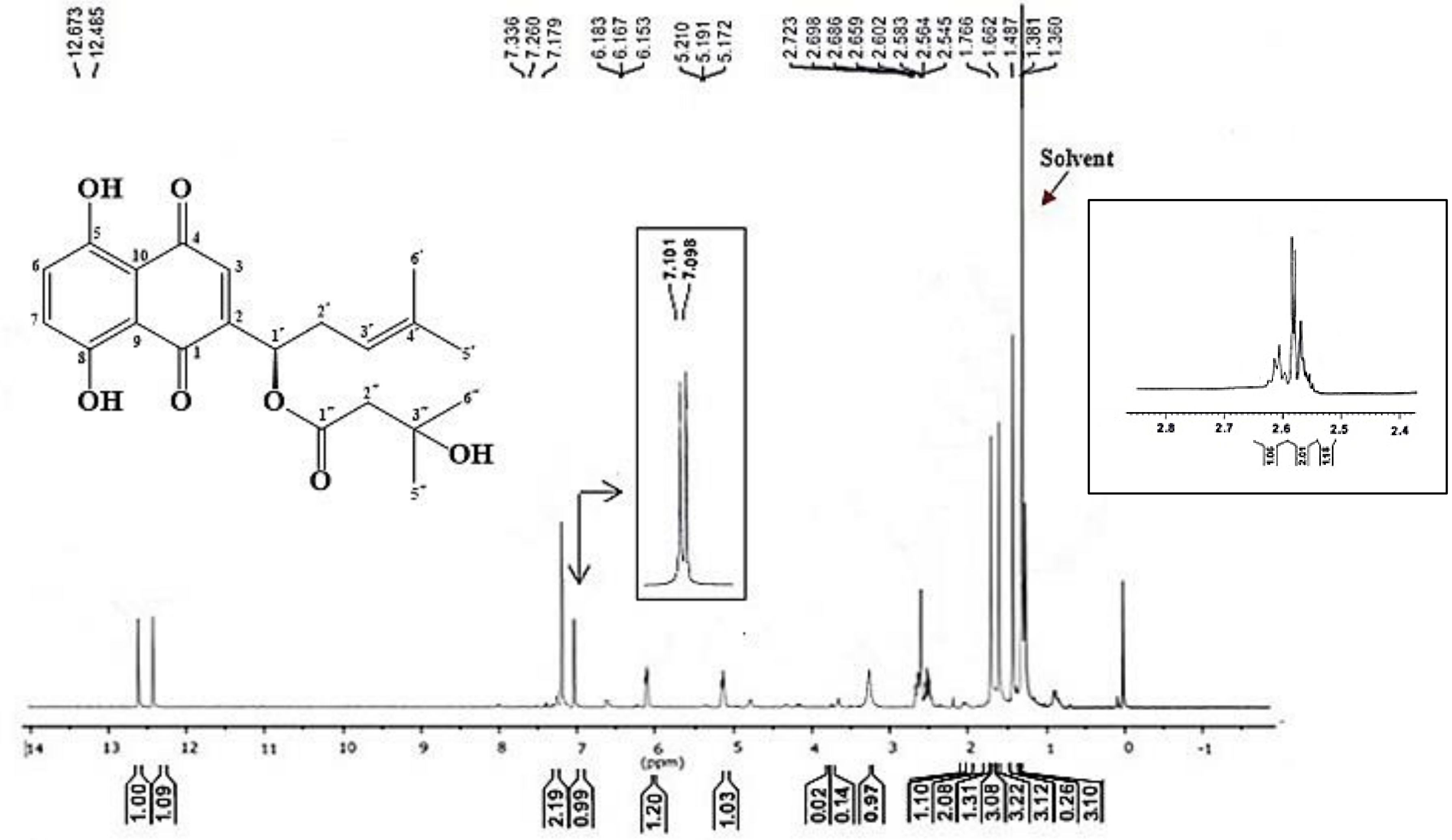

Figure S6. ^{1}H NMR spectrum of compound **F.F4 (**$CDCl_3$, 400 MHz**)**

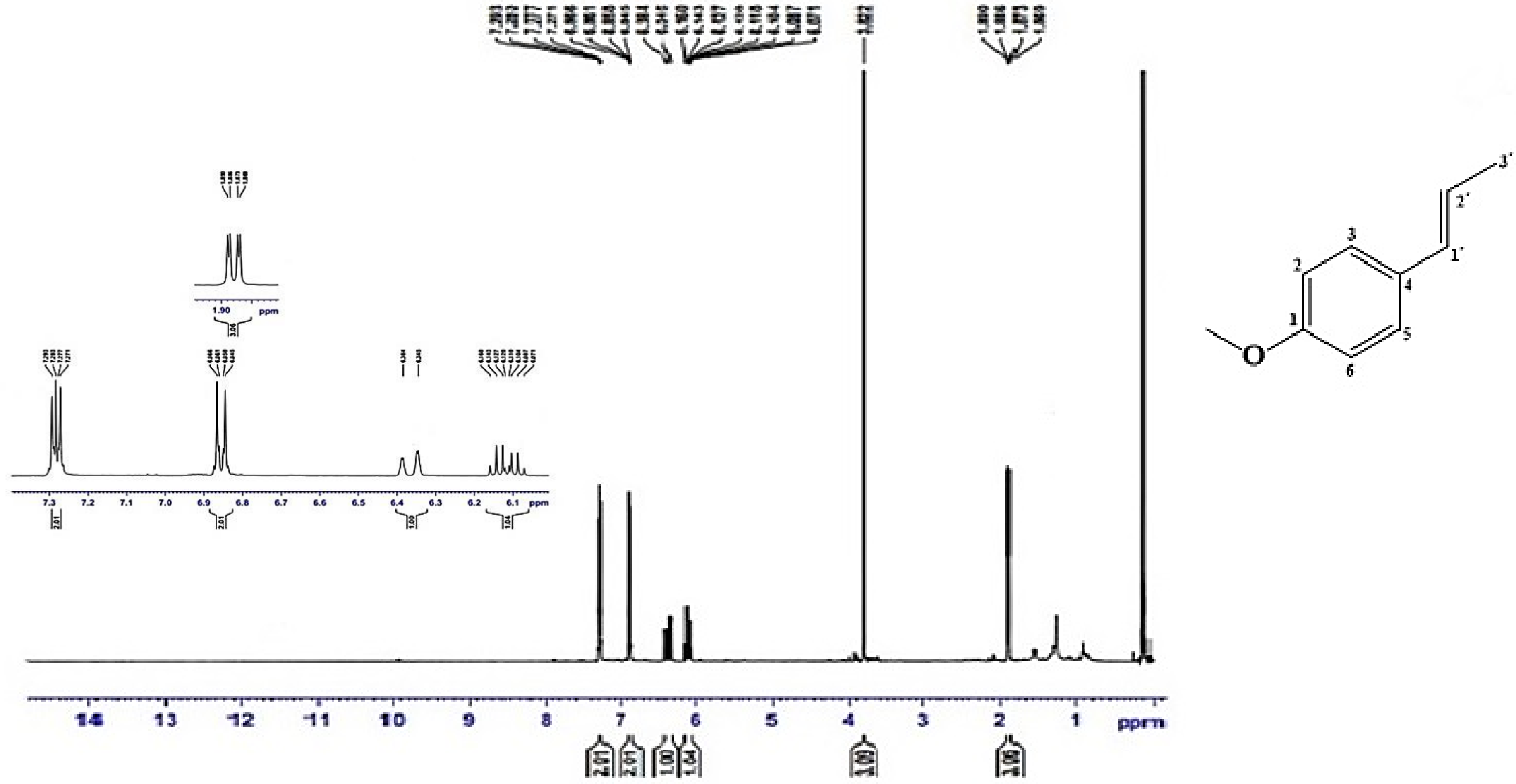

Figure S7. ^{1}H NMR spectrum of compound **F.F$_5$ (**$CDCl_3$, 400 MHz**)**
